# Nonpharmacological approaches for pain and symptoms of depression in people with osteoarthritis: systematic review and meta-analyses

**DOI:** 10.1038/s41598-023-41709-x

**Published:** 2023-09-18

**Authors:** Claire V. Burley, Anne-Nicole Casey, Matthew D. Jones, Kemi E. Wright, Belinda J. Parmenter

**Affiliations:** 1 UNSW Medicine & Health Lifestyle Clinic, School of Health Sciences, University of New South Wales, Sydney, Australia; 2 Centre for Healthy Brain Ageing, School of Clinical Medicine, University of New South Wales, Sydney, Australia; 3 Department of Exercise Physiology, School of Health Sciences, University of New South Wales, Sydney, Australia; 4 Centre for Pain IMPACT, Neuroscience Research Australia, Sydney, Australia

**Keywords:** Osteoarthritis, Ageing, Depression

## Abstract

People with osteoarthritis often experience pain and depression. These meta-analyses examined and compared nonpharmacological randomized controlled trials (RCTs) for pain and symptoms of depression in people living with osteoarthritis. RCTs published up until April 2022 were sourced by searching electronic databases EMBASE, PUBMED & MEDLINE, Web of Science, CINAHL and PEDro. Random-effects meta-analyses were performed to calculate pooled effect sizes (ES) and 95% confidence intervals (CI) for pain and depression. Subgroup analyses examined intervention subtypes. For pain, 29 interventions (n = 4382; 65 ± 6.9 years; 70% female), revealed a significant effect on reducing pain (ES = 0.43, 95% CI [0.25, 0.61], *p* < 0.001). Effect sizes were significant (*p* < 0.001) for movement meditation (ES = 0.52; 95% CI [0.35, 0.69]), multimodal approaches (ES = 0.37; 95% CI [0.22, 0.51]), and psychological therapy (ES = 0.21; 95% CI [0.11, 0.31]), and significant (*p* = 0.046) for resistance exercise (ES = 0.43, 95% CI [− 0.07, 0.94]. Aerobic exercise alone did not improve pain. For depression, 28 interventions (n = 3377; 63 ± 7.0 years; 69% female), revealed a significant effect on reducing depressive symptoms (ES = 0.29, 95% CI [0.08, 0.49], *p* < 0.001). Effect sizes were significant for movement meditation (ES = 0.30; 95% CI [0.06, 0.55], *p* = 0.008) and multimodal interventions (ES = 0.12; 95% CI [0.07, 0.18], *p* < 0.001). Resistance/aerobic exercise or therapy alone did not improve depressive symptoms. Mind–body approaches were more effective than aerobic/resistance exercise or therapy alone for reducing pain and depression in people with osteoarthritis.

**Systematic review registration:** PROSPERO CRD42022338051.

## Introduction

Arthritis and psychological conditions (i.e. mental health conditions, for example, depression and anxiety) were in the top three most prevalent chronic conditions experienced in Australia during 2020–2021^1^. Osteoarthritis (OA) is a degenerative joint condition where cartilage that cushions the ends of the bones deteriorates. It is the most common form of arthritis and most commonly affects hands, knees, hips and lower back and neck. Depression and anxiety are both common psychological co-morbidities that occur alongside OA^2,3^ in addition to increased pain and other mental health issues^4^. Chronic pain is experienced by one in five Australians aged 45 and over^5^.

OA has also been associated with increased dementia risk^6,7^, higher prevalence rates of dementia^8^, and is considered an early risk factor for cognitive decline^7^. Depression is another risk factor for dementia^9^ and is a common psychological symptom associated with dementia^10^. People living with dementia often experience other behavioural changes and psychological symptoms (e.g. agitation) that may be associated with underlying pain that is not always identified by care staff due to communication difficulties^11^. In Australia, dementia is the leading cause of death in women and second leading cause of death following heart disease in all Australians^1,12^. High rates of OA and dementia are also observed globally^13^.

There are no medications that can cure OA, though analgesics, non-steroidal anti-inflammatory drugs (NSAIDs) and topical therapies are often administered to relieve pain and inflammation. However, all medicines have side effects, for example, NSAIDS can increase risk of stomach ulcers and bleeding^14^, paracetamol can have adverse side effects when taken long-term^15^, and the use of opioids is controversial, with increasing evidence showing an increase in addiction, morbidity, and mortality^16^. Therefore, effective nonpharmacological interventions are needed to address pain and depression in OA, which are all well-established precursors to dementia. No previous systematic reviews have sought to determine the most effective approaches for reducing pain levels and symptoms of depression in OA. Reducing these co-morbidities will reduce dementia risk.

Physical and psychological health conditions rarely occur in isolation, and it is more likely people will go onto experience multiple comorbidities. For example, co-morbidities including psychological conditions are more likely in those with pain than those without^5^. The ABS reported that in 2020, 19% of Australians with long-term health conditions had two or more chronic conditions^1^. The interactions between physical and psychological conditions and associated symptoms are complex, and conditions may present independently or be highly intertwined. Common brain-related conditions (e.g. depression, anxiety, and dementia) can be exacerbated by physical conditions (e.g. OA, cardiovascular disease (CVD)) and vice-versa.

There are several models/theories that describe proposed mechanisms involved in these interactions. The biopsychosocial model which recognizes the contribution of all biological, psychological, social, and behavioural factors that dynamically interact with one another to generate the experience of pain^17^. Inflammation models describe how localized peripheral inflammation can trigger the development of neuroinflammation and subsequently the induction of dementia pathology^18^. The pain-sensitisation model recognises highly variable experiences of pain between individuals and the role of neuroplastic changes occurring in the peripheral and central nervous system, resulting in pain sensitisation, and impacting the person’s experience of pain^19^. The fear-avoidance model of musculoskeletal pain describes a psychological process where concern or fear about pain can lead to fear-avoidance behaviour which paradoxically can exacerbate pain and function^20^. The role of the gut microbiome has also been implicated with pain in OA^21^, depression^22,23^ and neurological conditions^24^.

There are clear associations between physical, psychological, and cognitive health outcomes (i.e. OA, anxiety, depression, and cognitive decline). However, it is not yet known what the most effective approaches are for improving psychological outcomes as well as physical outcomes (e.g. pain) in people living with OA. Physical activity interventions delivered by Accredited Exercise Physiologists (AEPs) have been shown to be highly effective for people living with 26 chronic conditions^25^, including OA^26,27^, anxiety and depression^28,29^, cognitive decline/dementia^30,31^, diabetes^32^ and cardiovascular disease^33^. Examples of effective exercise strategies include resistance training/strengthening exercises^34^, yoga^35^, and aquatic exercise^36^. Interventions that include lifestyle education and information on managing chronic conditions (e.g. information specific to OA, diabetes, depression, etc.) also provide additive beneficial effects^37^. Indeed, exercise and education are key components of clinical guidelines for OA. It is important that clients understand how to self-manage their pain and remain engaged with exercise to improve outcomes.

Research shows that therapeutic approaches such as cognitive behavioural therapy (CBT) are highly effective in treating mild-moderate depression and anxiety^38^. Furthermore, evidence suggests that the combination of exercise and behavioural therapies provides greater reduction in depressive symptomology than therapy alone^39,40^. Behavioural therapy approaches have been adapted or investigated in specific populations such as diabetes^41^, sleep disorders^42^, eating disorders^43^, and chronic pain^44^. Multimodal approaches that include a combination of exercise, therapeutic approaches and/or lifestyle education may be more effective than single mode approaches due to the focus on both psychological and physical health outcomes. However, there is limited evidence around the effect of multimodal treatment on multimorbidity.

Therefore, the aims of this systematic review and meta-analysis are to determine the most effective nonpharmacological approaches for reducing pain and symptoms of depression in people living with OA. We aimed to:

1. Identify effective approaches for improving pain and reducing symptoms of depression in people with OA.
2. Identify whether single or multi-modal approaches are more effective.
3. Present evidence-based recommendations for treatments for people with OA and associated depression.

In better understanding how to reduce OA-related symptoms and co-morbidities in populations who are at higher-risk of experiencing cognitive decline (i.e. people living with OA^6^), we may also reduce dementia prevalence rates, as well as improve psychological outcomes for people living with OA.

## Methods

The review was preregistered with the international prospective register of systematic reviews (PROSPERO), in accordance with PRISMA guidelines (PROSPERO CRD42022338051)^45^.

### Search strategy

Relevant studies published from electronic database inception until April 2022 were sourced by searching: EMBASE, PUBMED & MEDLINE, Web of Science, CINAHL and PEDro. Studies that included outcome measures relevant to physical outcomes (e.g. pain and physical function) and psychological outcomes (i.e. depression and anxiety symptomatology) were included in the initial search and this review. Search terms were Osteoarthritis OR OA; Depression OR anxiety OR mood OR affective OR psychological; Exercise OR physical activity OR education OR therapy OR CBT OR cognitive behavioural OR cognitive-behavioural OR lifestyle intervention OR mindfulness; RCT OR Randomised controlled trial OR Randomized controlled trial OR randomised controlled trial OR Randomized controlled trial (see Supplementary Material S1 for full list of search terms).

### Eligibility criteria and study selection

Only randomized controlled trials (RCTs) were included in this review. Studies were included from all settings such as community clinics, hospitals, and in people’s homes via online platforms, if pain or symptoms of depression in people with OA were assessed. Studies were only included where two or more conditions were compared (control versus intervention). Exclusion criteria were studies having an active control group (e.g. aerobic activity versus resistance activity), studies where data was not presented separately for people with OA if they included people with other pain-related conditions (e.g. lower back pain), studies not available in English, and studies with insufficient information to conduct a meta-analysis. When means and standard deviations were not provided, authors were contacted and requested to share this data.

Only post-intervention scores were compared across conditions (i.e. post-intervention, control versus intervention group scores in pain and symptoms of depression). Cochrane methods and those developed by statisticians were used to convert data^46–48^. Pooled, weighted effect sizes were calculated for all approaches, followed by subgroups determined by intervention types (e.g. multimodal, aerobic activity only, resistance activity only, therapeutic only, movement meditation).

### Interventions

Only nonpharmacological interventions were included that involved either exercise, lifestyle education, therapeutic and/or multimodal approaches. These were categorized into groups as they emerged and refined during the screening process. The final groups were ‘multimodal approaches’, ‘movement mediation’ (including yoga, tai chi, and chi gong), ‘resistance activity only’, ‘aerobic activity only’, and ‘therapeutic approaches only’. Studies were included if they had one or more active interventions and an inactive control group (e.g. treatment as usual). Only data from the combined intervention and control group were used for the meta-analyses.

### Outcome measures

Studies were included that used several different measurement tools to determine pain and depression in people living with OA. For pain these included the Western Ontario and McMaster Universities Arthritis Index (WOMAC)^49^, Knee Injury and Osteoarthritis Outcome Score (KOOS)^50^, Hip Disability and Osteoarthritis Outcome Score (HOOS)^51^, 36-Item Short Form Survey (SF-36) (pain component)^52^, (PISF), Harris Hip Score (HHS) (pain component)^53^, and arthritis self-efficacy (ASE) (pain component)^54^. For depression these included the Centre for Epidemiological Studies Depression (CESD) scale^55^, Hospital Anxiety and Depression Scale (HADS)^56^, Geriatric Depression Scale (GDS)^57^, Beck Depression Inventory (BDI), (AIMS) (psychological component)^34^, Emotional Distress and Depression Short-Form (EDD-SF)^58^, Depression Anxiety Stress Scales (DASS21)^59^, and the Patient Health Questionnaire (PHQ-9)^60^. Studies were included with a range of intervention durations and some included follow-up periods (e.g. 6 months, 1-year) which were considered when rating of the quality of the research article was completed. Articles were only included if they were in English.

### Quality rating

All studies were quality rated based on the study design, participant characteristics, outcome measures and statistical analyses using a Quality Rating Tool^10^ (see Supplementary Material S2) developed by adapting and combining several different previously used tools^10,61,62^. The tool ensured quality ratings reflected ability to determine potential statistical and clinical significance. Points were given based on study design and statistical analysis. Total scores on the quality rating tool ranged from 0 to 16 (i.e. higher scores indicated higher quality research).

### Statistical analysis and clinical interpretation

The statistical analysis was conducted in Meta-Essentials developed by Suurmond and colleagues, Netherlands^63^. A random effects meta-analysis was completed with restricted maximum likelihood estimation (REML), assuming that the true effects differ across studies, as these studies vary in their study design and methodology (e.g. intervention type, duration, and outcome measure). Sensitivity analyses were performed to determine potential studies driving heterogeneity. Subgroup analyses were performed to investigate different types of interventions on clinical outcomes (pain and depression) and associated significance and effect sizes. The effect size of interventions was determined using Hedges’ *g*^64^ (page 110). Bias corrected standardized mean difference calculations (Hedges’ *g*) are recommended for studies with small sample sizes. According to Cohen, effect sizes less than 0.2 are trivial, 0.2 are small, 0.8 are medium, and 0.8 or higher are large^65^. Evidence for minimally clinically important differences (MCID) and moderate improvement estimates of change in OA-related pain had been published^66^. For the KOOS and HOOS short form pain measures, evidence has been published for MCID and moderate improvement estimates (2.2 and 15 respectively). Though reliability was much lower in knee compared to hip OA^66^.

A 95% confidence interval was used to determine the efficacy of combined interventions versus control on pain and symptoms of depression. Heterogeneity of the effects was assessed by calculating the I^2^ index and prediction intervals. The I^2^ index value reflects the proportion of true heterogeneity in the observed variance 69, 47], where 0% indicates no observed heterogeneity and increased heterogeneity is indicated by larger values where 25%, 50% and 75% reflect low, medium, and high heterogeneity respectively^67^. Prediction intervals are also presented throughout to ensure the predicted range of effects is unambiguous^68^. To understand the observed heterogeneity, we performed subgroup analyses to test intervention type. Publication bias was assessed graphically with contour-enhanced funnel plots and Egger’s regression test^69^. Funnel plots show if studies were missing only from areas of low statistical significance indicating that any asymmetry is likely to be caused by publication bias. The Egger regression gives “the degree of funnel plot asymmetry as measured by the intercept from regression of standard normal deviates against precision”^69^. If Egger’s test results in a *p* value less than 0.05, this implicates publication bias.

## Results

### Included studies

**Figure 1:**
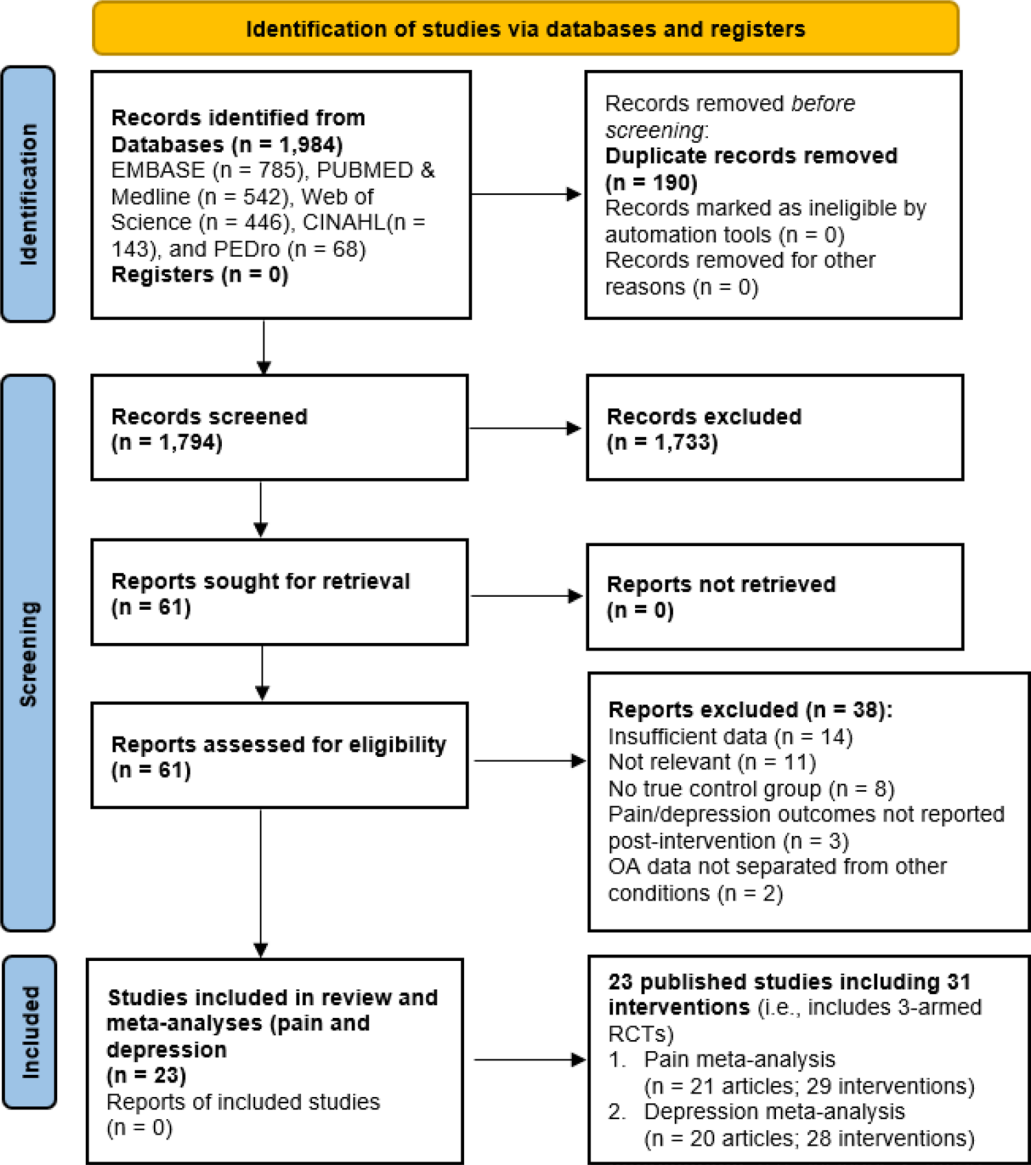
PRISMA flow diagram.

In total 1984 studies were found through the database search of studies published up until April 2022 (Fig. 1). Following removal of duplicates, article screening and removal of studies that did not meet eligibility criteria, 61 studies remained that included measures of pain and/or symptoms of depression in people with OA (Fig. 1). Thirty-eight studies were then excluded for not having sufficient data for meta-analysis, an active control condition, pain or depression outcomes not being reported post-intervention, and articles that did not meet methodology requirements (i.e. not RCTs, protocol papers). The mean quality rating of the 23 remaining studies (see Table 1) used in the final meta-analyses was high (mean: 13; SD = 1.6; total possible score: 16; range: 10–16). Participant characteristics are outlined in Table 2.

**Table 1:** Nonpharmacological approaches for pain levels and symptoms of depression in people with osteoarthritis (31 interventions from 23 studies).

Author, year, country, setting	Interventions (description, frequency and duration)	Study design (randomisation method, number of participants, OA site/s, depression severity, blinding, follow-up)	Outcomes & measures (effect size; Hedge’s *g*)	Quality rating
Aerobic exercise only (4 interventions from 4 studies)				
*Cheung et al. 2017, USA^70^, Community and home	A low-impact aerobic and strengthening exercise program that involved 15 min of mild aerobic exercise for a full-body warm-up, 30 min of strengthening exercises (isometric and isotonic exercises, i.e. without/with moving the joints). Participants were also asked to practice the aerobic portion 15–30 min/day and the strengthening portion 30 min/day on non-consecutive days at home. The program was progressive in nature 8 weeks: One weekly 45-min group session with 2–4 days per week home practice sessions	Three-armed RCT (Aerobic strengthening, hatha yoga & control) Raters were blinded No post-intervention follow-up	Pain levels: WOMAC *d* = 0.00 Depression: HADS *d* = − 0.12	13
*French et al. 2013, Dublin^71^, Academic teaching hospitals	Exercise therapy (ET) sessions were administered by a senior grade or clinical specialist physiotherapist. They included flexibility and strengthening exercises delivered using a semi-structured protocol. The protocol provided guidance on exercise prescription and progression but could be tailored to individual patient physical assessment findings. Strengthening focused on low-load exercise, commencing in non-weight-bearing positions, and progressing to functional positions. The key target muscles were the gluteal muscles. A daily home exercise program supplemented the clinic-based treatment. Participants were also encouraged to undertake aerobic exercise, such as walking, cycling, or swimming for at least 30 min, 5 days a week, and were given written and verbal information on the principles of aerobic conditioning, such as pacing, gradually progressing intensity and time of exercise, and incorporating exercise into daily life 8 weeks: One 30-min session per week (8 sessions in total)	Three-armed RCT (exercise therapy, ET with adjunctive manual therapy, control) Raters were blinded No post-intervention follow-up	Pain levels not reported Depression: HADS *d* = 0.16	15
*Kuntz et al. 2018, Canada^34^, University setting	The traditional exercise (TE) intervention reflected the current gold standard of strengthening exercise for knee OA. The program emphasized knee strengthening but also involved an aerobic warm-up, balance exercises, and stretching. TE was designed and supervised by kinesiologists and physical therapists and took place at a physical activity center. The sessions involved a ten-minute warm-up performed on a treadmill or cycle ergometer. Then, lower extremity strengthening was performed on pneumatically resisted exercise machines. Exercises included all major muscle groups of the lower extremity. The quadriceps were targeted at every session. Participants also completed balancing activities and static stretching. There was a progressive increase in the number of sets and resistance during strengthening exercises over the course of the intervention 12 weeks: 1-h, 3 times a week (36 sessions in total)	Three-armed RCT (Traditional exercise, bio-mechanically-based yoga & control) Raters were blinded No post intervention follow-up	Pain levels: KOOS *d* = 0.75 Depression: CESD *d* = − 0.09	13
Lin et al. 2020, Taiwan^72^, Hospital clinic	The active video games treatment involved using a computerised system called “Hot Plus” (Supreme Investment Co., Taipei). Two sessions were selected that required participants to move their trunk and lower limbs as fast as possible to tread on the step-sensing pad placed on the floor to complete on-screen tasks. The first session was ‘‘Whack-a-mole,’’ which required the player to stump moles into the holes when virtual moles appeared. It had 3 different levels. More and faster moles appeared simultaneously at higher difficulty levels. The second session was ‘‘Archery,’’ which required the player to step on the sensing pad to shoot an arrow when virtual dynamic targets appeared on the screen. This task also had 3 difficulty levels. Faster and smaller shooting targets appeared on the screen at higher difficulty levels 4 weeks: 20 min, 3 times a week (12 sessions in total)	Two-armed RCT (active video games versus traditional therapeutic exercise Raters were blinded One- and three-months post intervention follow-up	Pain levels: WOMAC *d* = 0.05 Depression: HADS *d* = − 0.03	12
Movement meditation (7 interventions from 6 studies)				
*Cheung et al. 2017, USA^70^, Community and home	The hatha yoga program was designed by a group of expert yoga teachers. Sessions included poses in the seated, supine, prone, and standing positions; breathing exercises; and relaxation/mindfulness training. Key yoga poses included “easy” seated pose, reclining bound angle, half locust variation, head to knee pose, warrior I and II, tree pose variation, bridge, reclining hamstring stretch with hip opener with strap, reclining twist and relaxation pose. A progressive series of poses with props such as yoga mats, blocks, straps, blankets, and chairs were used, and poses were modified when needed based on the participant’s physical abilities to increase confidence and the ability to remain in the pose and receive the benefits. Each yoga class consisted of 8–10 yoga poses with 2–3 new variable poses introduced at each session. A registered yoga instructor who was experienced in working with older adults with functional limitations taught the classes 8 weekly 45-min group classes with 2–4 days per week home practice sessions	Three-armed RCT (Hatha yoga, aerobic strengthening & control) Raters were blinded No post-intervention follow-up	Pain levels: WOMAC *d* = 0.51 Depression: HADS *d* = -0.32	13
*Fransen et al. 2007, Australia^27^, Community	Tai chi sessions were facilitated by four different Tai Chi instructors trained in a special program for arthritis designed by the study author Paul Lam. This program is a modification of 24 forms from the Sun style of Tai Chi and includes a preliminary 10-min warm-up session. Participants were able to purchase, if they desired, a Tai Chi video to assist with home practice 12 weeks: 1-h, 2 times a week (24 sessions in total)	Three-armed RCT (Tai chi, hydrotherapy, waiting-list control) Raters were blinded 6-month post intervention follow-up	Pain levels: WOMAC *d* = 0.52 Depression: DASS-21 *d* = 0.21	14
*Kuntz et al. 2018, Canada^34^, University setting	The biomechanically based yoga exercise program was led by a certified, trained yoga instructor and consisted of alignment-based postures that activate the lower limb musculature while maintaining a low knee abduction movement. The selected weight-bearing, static poses were performed barefoot and included squats and lunges with varying foot, trunk, and arm positioning. Careful attention was given to ideal alignment of the leg throughout the exercises. The classes began with a body-awareness exercise performed in supine followed by the strengthening postures and concluded with a closing deliberate relaxation exercise performed in supine. Exercise difficulty was progressively increased 12 weeks: 1-h, 3 times a week (36 sessions in total)	Three-armed RCT (Traditional exercise, bio-mechanically-based yoga & control) Raters were blinded No post intervention follow-up	Pain levels: KOOS *d* = 0.99 Depression: CESD *d* = 0.51	13
Moonaz et al. 2015, Canada^35^, Hospital affiliated fitness centres	The yoga program was designed by a registered yoga therapist (SM) with input from Johns Hopkins Arthritis Center faculty. Two yoga therapists with 10 + years of experience taught classes. Each class began with questions/comments (5 min), breathing exercises and chanting (5 min), warm-up and moving sequence (surya namaskara; 15 min), and isometric poses (asanas) (20 min) to increase strength, flexibility and balance. Classes ended with deep relaxation (sivasana; 10 min), a closing chant, and meditation (5 min). See supplemental file for sample class and modifications. Poses included gentle forward bends, backbends, twists, balances, standing, sitting, and lying poses, and were modified for individual at the discretion of the teacher and/or participant. Complexity of poses and intensity was standardized to allow gradual progression 8 weeks: 1-h, 2 times a week (16 sessions in total)	Two-armed RCT (yoga versus wait-list control) Raters were blinded 9-month post intervention follow-up	Pain levels: SF-36 (pain component) *d* = 0.62 Depression: CESD *d* = 0.84	13
*Park et al. 2016, USA^73^, Community centres	English linguistically tailored yoga program. Sit ‘N’ Fit Chair Yoga, designed for older adults with OA, is performed while sitting in a chair with arms, for easy access and standing. The chair is used for support for the standing poses. The intervention was developed by a research team of health care providers with a yoga teacher who has taught yoga for more than 15 years and is certified by the International Yoga Alliance. The yoga intervention consists of four components while using the support of a chair: breath of life (10 min), body proper (20 min), warrior in the body (5 min), and mind–body connection (10 min) 8 weeks: Two 45-min sessions per week (16 sessions in total)	Four-armed RCT (chair yoga—English, chair yoga – Spanish, English/Spanish control: health education program) Raters were blinded One- and three-month post-intervention follow-up	Pain levels: WOMAC *d* = 0.19 Depression: EDD-V1 *d* = 0.12	12
*Park et al. 2016, USA^73^, Community centres	Spanish linguistically tailored yoga program. Sit ‘N’ Fit Chair Yoga, designed for older adults with OA, is performed while sitting in a chair with arms, for easy access and standing. The chair is used for support for the standing poses. The intervention was developed by a research team of health care providers with a yoga teacher who has taught yoga for more than 15 years and is certified by the International Yoga Alliance. The yoga intervention consists of four components while using the support of a chair: breath of life (10 min), body proper (20 min), warrior in the body (5 min), and mind–body connection (10 min) 8 weeks: 45-min, 2 times a week (16 sessions in total)	Four-armed RCT (chair yoga – Spanish, chair yoga – English, English/Spanish control: health education program) Raters were blinded One- and three-month post-intervention follow-up	Pain levels: WOMAC *d* = 0.83 Depression: EDD-V1 *d* = 0.43	12
Zhang et al. 2022, China^74^, Hospital	Traditional Chinese Yijinjing Qigong exercise provided by professional instructors. Each session included a 5-min warm-up and cool-down period and a 30-min practice. Phase 1 (weeks 1–2) focused on fundamental principles, movement techniques, safety precautions. Phase 2 (weeks 3–4) focused on learning/practicing forms/associated movements. Phase 3 (weeks 5–12) involved completion of the family exercise plan, review video/homework materials 12 weeks: 40 min, 2 times a week (24 sessions in total)	Two-armed RCT (qigong, stretching control) Raters were blinded (single-blinded) One- and three-month post intervention follow-up	Pain levels: WOMAC *d* = 0.42 Depression: BDI *d* = 2.57	14
Multimodal (10 interventions from 8 studies)				
*Ahn and Ham 2020, South Korea^75^, Community	Experimental group 1 – Program outlined below with muscle strengthening plus stretching Health education and counselling combined with exercise classes based on the Interaction Model of Client Health Behaviour. The intervention involves 8 sessions and promotes cognitive-affective-behavioural skills including education and counselling on the disease characteristics and treatment options, encouraging self-responsibility, strengthening self-efficacy, self-monitoring, positive reinforcement, and emotional support regarding pain control, medication adherence, depression, and diet. Communication skills training was also provided to promote the client-professional interaction. Trained community health nurse practitioners led the intervention Duration not clear: 8 sessions (4 individual and 4 group), individual home visits were 30–60 min and group community sessions were 60–90 min	Three-armed RCT (multimodal approach with muscle strengthening plus stretching, multimodal approach with muscle strengthening plus walking, control) Raters were blinded No post intervention follow-up	Pain levels: WOMAC *d* = 0.66 Depression: CESD *d* = 0.07	13
*Ahn and Ham 2020, South Korea^75^, Community	Experimental group 2 – Program outlined above with muscle strengthening plus walking Duration not clear: 8 sessions (4 individual and 4 group), individual home visits were 30–60 min and group community sessions were 60–90 min	Three-armed RCT (multimodal approach with muscle strengthening plus walking, multimodal approach with muscle strengthening plus stretching, control) Raters were blinded No post intervention follow-up	Pain levels: WOMAC *d* = 0.78 Depression: CESD *d* = 0.11	13
Barlow et al. 2000, UK^76^, Community (telephone-based)	An RCT of the arthritis self-management programme (ASMP). ASMP is multicomponent and topics included: information about arthritis, an overview of self-management principles, exercise, cognitive symptom management (e.g. distraction, visualization, guided imagery), dealing with depression, nutrition, communication with family and health professionals, and contracting (setting of realistic goals during the forthcoming week). Participants report back to their group on their achievements at the weekly session. Participants are given a copy of The Arthritis Help Book (Lorig & Fries, 1995). Interactive course with group discussion, problem solving, role plays and mastery experience (trying out skills introduced) 4 months: 6 weekly 2-h sessions, delivered by pairs of lay leaders (most with arthritis)	Two-armed RCT (self-management program versus waitlist-control) Unclear whether the raters were blinded 12-month post intervention follow-up	Pain levels: ASE *d* = 0.31 Depression: HADS *d* = 0.12	11
*French et al. 2013, Dublin^71^, Academic teaching hospitals	Exercise therapy (ET) with adjunctive manual therapy (MT) The sessions included ET (as described above) and up to 15 min of MT in line with current clinical practice at participating sites. A choice of nonmanipulative MT techniques based on pain/stiffness relations and movement restrictions of the affected hip was available, with no more than 5 MT techniques allowed during an individual session 8 weeks: One 45-min session per week (8 sessions in total)	Three-armed RCT (ET + MT, ET alone, control) Raters were blinded No post-intervention follow-up	Pain levels not reported Depression: HADS *d* = 0.18	15
Hurley et al. 2007, UK^77^, Primary care providers	Rehabilitation program integrating exercise, self-management, and active coping strategies. The rehabilitation program combined discussion on specific topics regarding self-management and coping, etc., with an individualized, progressive exercise regimen. To ensure consistency in content and delivery, the same experienced physiotherapist devised, supervised, and progressed all sessions for all participants 6 weeks: Twice weekly (12 sessions in total)	Two-armed RCT (rehabilitation program, usual care) Raters were blinded 6-month post intervention follow-up	Pain levels: WOMAC *d* = 0.29 Depression: HADS *d* = 0.16	13
*Somers et al. 2012, USA^78^, Community and clinic	Lifestyle behavioural weight management (BWM) only. Each participant was given a copy of a manual, which focused on 5 elements related to weight loss: lifestyle, exercise, attitudes, relationships, and nutrition, and contains 16 weekly sessions. The content of most of the group sessions was based on the weekly topic(s). In addition, participants received Appetite Awareness Training for 2 sessions, which emphasized the importance of attending to internal hunger and fullness cues. The overall goal of the program was a weight loss of 1–2 pounds a week achieved by gradually decreasing calorie and fat intake through permanent lifestyle changes 24 weeks: One 60-min sessions per week for first 6 months, One 60-min session every other week for the second 6 months	Four-armed RCT (PCST, BWM, PCST + BWM, standard care) Raters were blinded 4-weeks post intervention follow-up	Pain levels: WOMAC *d* = 0.17 Depression: AIMS (psychological subscale) *d* = 0.00	14
*Somers et al. 2012, USA^78^, Community and clinic	Pain coping skills training (PCST) plus lifestyle behavioural weight management (BWM). Participants concurrently received the BWM protocol described above and the PCST protocol described under the therapeutic section of this table. During the first 12 weeks, participants attended 120 min of group sessions that first presented the BWM protocol and then the PCST protocol. During the first 12 weeks, participants also attended three 90-min supervised exercise sessions each week. During the last 12 weeks, participants attended 120 min of group sessions held every other week that first presented the BWM protocol and then the PCST protocol. All PCST + BWM groups were delivered by clinical psychologists referenced above 24 weeks: One 60-min sessions per week for first 6 months, One 60-min session every other week for the second 6 months	Four-armed RCT (PCST, BWM, PCST + BMW, standard care) Raters were blinded 4-weeks post intervention follow-up	Pain levels: WOMAC *d* = 0.72 Depression: AIMS (psychological subscale) *d* = 0.35	14
Tak et al. 2005, Netherlands^79^, Community	'Hop with the Hip’ exercise program with strength training and lifestyle advice versus self-initiated contact with their general practitioner (usual care). The program included strength training sessions using fitness equipment under supervision of a PT, OA education from PT, guidance for a home exercise program, personal ergonomic advice (given by an OT), education on dietary aspects (given by a dietician) and participants with BMI > 30 given personal consultation. Further information was also available via a special telephone line 8 weeks: one 1-h group session per week (8 sessions in total)	Two-armed RCT (program, control) Raters were blinded 3-months post intervention follow-up	Pain levels: Harris Hip Score (HHS) pain subscale *d* = 0.52 No depression measures	10
Walsh et al. 2020, UK^80^, Community (physio. clinic)	The Facilitating Activity and Self-management in Arthritic Pain (FASA) program is a group exercise and self-management intervention facilitated by a physiotherapist. The intervention included approximately 20–25 min of group discussion and problem-solving session (with a supporting handbook) regarding issues of self-management. Topics included activity-rest cycling, use of ice and heat for pain relief, goal setting and action plans, exercise recommendations, healthy eating and managing changes in pain. After each discussion, participants undertook approximately 30–35 min of exercise, based on stations of strengthening, aerobic and co-ordination activities. Further to the exercises, each individual completed an action plan regarding exercise/activities they aimed to achieve over the following week 6 weeks: 60-min 2 times per week (12 sessions in total)	Two-armed RCT (program, control) Raters were blinded 6-months post intervention follow-up	Pain levels: DI-SMFA *d* = 0.13 Depression: HADS *d* = 0.07	16
Yip et al. 2008, Hong Kong^81^, Hospital out-patient clinic and a Telehealth wellness centre	Modified Arthritis Self-Management Programme (ASMP: Lorig et al. 1985)^82^ with an added exercise component. Small group classes (10–15 people) were led by registered nurses trained in leading small groups and self-management principles, standard ASMP plus goal-directed exercise component relevant to the group’s lifestyle habit: stretching exercises, walking, and Tai Chi types of movement – fluid, gentle, relaxed, slow – aimed at enhancing exercise for affected joints (Yip et al. 2007)^83^. The group practised the stretching exercise together twice each session, Tai Chi exercises were taught and reinforced for 30 min each session, intervention group were given pedometer for 3 days at baseline to reinforce walking. Routine conventional treatment prescribed by orthopaedic doctor or out-patient clinic was also provided 6 weeks: One 2-h session per week (6 sessions in total)	Two-armed RCT (ASMP, usual care) Raters were blinded Results are reported following 1-year post-initiation of an ongoing program	Pain levels: ASE (pain subscale; Lorig et al. 1985) *d* = 0.38 No depression measures	12
Resistance exercise only (3 interventions from 3 studies)				
Bossen et al. 2013, Netherlands^84^, Community (online in people’s homes)	Web-based physical activity intervention 'Joint2Move'. The program incorporates a baseline test, goal setting, time-contingent PA objectives (i.e. on fixed time points), and text messages to promote PA. Positive reinforcement is provided of gradual PA, despite the presence of pain. The gradual increase in activities changes the perception that PA is related to pain and reinforces confidence to improve PA performance. The Join2move intervention is a fully automated web-based intervention that contains automatic functions (web-based text messaging and automatic emails) without human support 9 weeks: self-paced program in which a patient’s favourite recreational activity is gradually increased in a time-contingent way	Two-arm RCT (web-based intervention, wait-list control) Raters were not blinded Three- and 12-month post-intervention follow-up	Pain levels: KOOS/HOOS *d* = 0.19 Depression: HADS *d* = 0.13	13
*Fransen et al. 2017, Australia^27^, Community	Hydrotherapy sessions were facilitated by four different registered physiotherapists. The hydrotherapy program (Fransen et al.^27^: Appendix A) was designed by the senior rheumatology physiotherapist 12 weeks: 1-h, twice a week	Three-armed RCT (Hydrotherapy, tai chi, waiting-list control) Raters were blinded 6-month post intervention follow-up	Pain levels: WOMAC *d* = 0.71 Depression: DASS-21 *d* = 0.50	14
Taglietti et al. 2018, Brazil^36^, Aquatic physiotherapy centre and primary health care unit	Aquatic exercise protocol in a heated pool, one-to-one sessions provided by certified physiotherapists. The water temperature was maintained at approximately 32 °C, with a depth of 1.2 m. The exercise protocol consisted of specific exercises: 5 min of warm-up with walking, patellar mobilization; stretching the leg muscles (quadriceps, gluteus, adductors and abductors of hip, triceps surae, and hamstrings); 15 min of knee and hip isometric and dynamic exercises with elastic bands (gluteus, adductors and abductors, quadriceps, hamstrings, and triceps surae); 20 min of aerobic exercises (stationary running or deep water-running); 10 min of step training and proprioceptive exercises; and 10 min of cool down with massage and relaxation 8 weeks: Two 60-min sessions per week (16 sessions in total)	Two-armed RCT (Hydrotherapy, education control) Raters were blinded Three-month post intervention follow-up	Pain levels: WOMAC *d* = 3.33 Depression: GDS *d* = 2.17	13
Therapeutic only (7 interventions from 7 studies)				
Allen et al. 2019, USA^85^, Community (telephone-based)	Pain coping skills training for African Americans. Culturally tailored pain telephone-based coping skills training (CST) program Coping skills training counselors provided instruction in cognitive and behavioral pain coping skills and led participants in guided rehearsals of these skills. Content included progressive muscle relaxation, mini-relaxation practices, communication with significant others about pain and coping, managing unhelpful mood, activity pacing, pleasant activities, pleasant imagery and other distraction techniques, physical activity and OA, weight management, problem solving and maintenance. Participants were asked to engage in home-based practice of the skills to enhance their application in pain-related situations. During each phone call, the counselor reviewed participants’ home practice, including successes and barriers, encouraged problem solving, and worked to set goals for application of skills. Participants were given handouts to facilitate each session, along with an audio recording to guide progressive muscle relaxation 3 months: 30–45 min sessions (11 sessions in total)	Two-armed RCT (intervention, wait-list control) Raters were blinded Three- and 9-months post intervention follow-up	Pain levels: WOMAS *d* = 0.17 Depression: PHQ-8 *d* = 0.10	16
Broderick et al. 2014, USA^86^	Pain coping skills training delivered by nurses. Training included cognitive and behavioural skills to manage pain and enhance self-perception of pain control. Four broad coping skills were taught across ten 30–45-min sessions: relaxation response, attention diversion techniques, altering activity and rest patterns as a way of increasing physical activity, reducing negative pain-related thoughts and emotions. The training included a treatment manual and home practice 10 weeks: One 30–45 min session per week (10 sessions in total)	Two-armed RCT (intervention, usual care) Raters were blinded Six- and 12-months post intervention follow-up	Pain levels: WOMAC *d* = 0.22 Depression: BDI *d* = 0.17	16
Hausmann et al. 2017, USA^87^, Academic Veterans Affairs Medical Centre	Positive psychology intervention program consisting of positive psychological skill-building activities drawn from the positive psychology literature and then refined based on qualitative input from patients. Included activities to build positive psychological skills focused on gratitude, kindness, optimism, mindfulness, self-affirmation, identifying and using personal strengths reflecting on good things, and forgiveness 6 weeks: 1 session per week (6 sessions in total)	Two-armed RCT (therapy, control) Raters were not blinded Three- and 6-months post intervention follow-up	Pain levels: WOMAC *d* = 1.08 Depression: PANAS *d* = 0.20	11
Helminen et al. 2015, Finland^88^, Community (groups)	Cognitive–behavioural group intervention (7 − 13 people) supervised by an experienced psychologist and a physiotherapist. Each session lasted for two hours with a 15 − 20 min break to enhance peer support and social bonding. The outline of the sessions included an introduction (15 min), lecture (knowledge and insight, max 15 min), problem solving (in pairs/teams, 15 − 20 min), skills training (15 − 20 min), homework assignments (15 min), and a résumé (feedback) of the session (15 min). A written example of a knee OA pain patient’s life was used throughout the intervention as a basis for discussion and practice in problem solving 6 weeks: One weekly 2-h session (6 sessions in total)	Two-armed RCT (therapy, control) Raters were blinded Three- and 12-months post intervention follow-up	Pain levels: WOMAC *d* = 0.18 Depression: BDI *d* = 0.02	15
Lin et al. 2003, USA^89^, Primary care clinics	Improving Mood-Promoting Access to Collaborative Treatment (IMPACT). Psychotherapeutic intervention using a collaborative care approach. Nurse/psychologist depression care management including psychosocial history, education, and behavioural activation, identify treatment preferences. Antidepressant medications and/or 6 to 8 sessions of psychotherapy (Problem-Solving Treatment in Primary Care). Usual care group received routinely available treatments including medication and referrals to speciality mental health services 1-year: 6–8 sessions	Two-armed RCT (therapy, usual care) Unclear whether the raters were blinded Three-, 6- and 12-months post intervention follow-up	Pain levels: RAND-36 (pain subscales) *d* = 0.18 Depression values not reported	14
O’Moore et al. 2018, Australia^90^, Community (online)	Internet Cognitive-Behavioural Therapy (iCBT Sadness Program). iCBT Sadness Program consists of lessons representing best practice CBT, as well as regular homework assignments and access to supplementary resources. Each lesson comprises a cartoon narrative in which a character gains mastery over symptoms of depression by learning and implementing CBT skills. Participants could submit queries via email or phone 10 weeks: 6 online sessions	Two-armed RCT (therapy, treatment as usual (TAU)) Raters were blinded Three-months post intervention follow-up	Pain levels: WOMAC *d* = 0.28 Depression: PHQ-9 *d* = 1.01	13
*Somers et al. 2012, USA^78^, Community and clinic	Pain coping skills training (PCST) only. PCST was delivered by clinical psychologists with prior PCST experience (1 to 5 years) who were systematically trained by a senior clinical psychologist who is an expert in pain coping skills training. Training included role-playing, listening to the protocol delivered on audiotape, and observation of PCST being delivered in a group format. Psychologists delivering the treatment for this protocol met for supervision weekly with the senior psychologist; audiotapes of sessions were reviewed to evaluate the concurrence between the session delivery and the intervention protocol and role-playing for the next session was conducted. Four psychologists led the PCST groups during the study. The PCST intervention was designed to 1) decrease maladaptive pain catastrophizing; and 2) enhance participants’ ability to control and decrease pain by increasing use of adaptive coping strategies (e.g. distraction, relaxation, and changing activity patterns) 24 weeks: One 60-min sessions per week for first 6 months, One 60-min session every other week for the second 6 months	Four-armed RCT (PCST, BWM, PCST + BWM, standard care) Raters were blinded 4-weeks post intervention follow-up	Pain levels: WOMAC *d* = 0.23 Depression: AIMS (psychological subscale) *d* = -0.12	14

*AIMS* Arthritis Impact Measurement Scale, *ASE* Arthritis Self-Efficacy scale, *BDI* Beck’s Depression Inventory, *CESD* Center for Epidemiological Studies – Depression, *DASS*-*21* Depression, Anxiety and Stress Scale – 21 items, *DI*-*SMFA* Dysfunction Index of the Short Musculoskeletal Functional Assessment, *EDD*-*v1* Emotional Distress and Depression—version 1, *GDS* Geriatric Depression Scale, *HADS* Hospital Anxiety and Depression Scale, *HHS* Harris Hip Score, *HOOS* Hip injury Osteoarthritis Outcome Score, *KOOS* Knee injury Osteoarthritis Outcome Score, *PHQ* Patient Health Questionnaire, *RAND*-*36* 36-item health survey, *PANAS* Positive And Negative Affect Schedule, *RCT* Randomised Control Trial, *SF*-36 Short Form survey (36-item), *WOMAC* Western Ontario and McMaster Universities Osteoarthritis Index.

*studies where more than one intervention have been included, e.g. 3-armed RCTs.

**Table 2:** Participant characteristics.

Author, year	N	Sex N and (%) female	Age in years [mean ± SD or median (IQR)]	Osteoarthritis (type and severity if known)	Diagnosed depression	Other characteristics
Ahn and Ham 2020^75^	90	80 (88)	71.3 ± 7.1	OA (not specified)	No	49% had hypertension, majority of participants were farmers
Allen et al. 2019^85^	248	122 (49)	59.0 ± 10.3	Hip/knee OA	No	None
Barlow et al. 2000^76^	544	168 (31)	I: 57.3 ± 13.2 C: 59.1 ± 12.3	OA (not specified)	No	OA condition duration in years mean ± SD: I: 13.9 ± 10.6 C: 13.6 ± 9.1 Arthritis type: 104 OA, 70 RA, 25 other
Broderick et al. 2014^86^	256	Not stated	I: 68.0 ± 8.7 C: 66.4 ± 10.3	Hip/knee OA Knee (vs. hip), 81%; 73% (of sample) Arthritis severity (K-L grading; I, C): 0 to 1; 17%, 27% > 1 to 2; 27%, 21% > 2 to 3; 26%, 23% > 3 to 4; 30%, 30%	No	OA condition duration in years mean ± SD: I: 10.7 ± 11.2 C: 11.3 ± 10.9 15–16% were receiving treatment for a psychiatric disorder and 10% had memory/thinking problems
Bossen et al. 2013^84^	199	129 (65)	I: 61 ± 5.9 C: 63 ± 5.4	Group: % of sample knee, hip, both OA I: 67%, 21%, 12% C: 60%, 20%, 19%	No	Number of comorbidities (I%, C%): None, (65, 60) One, (19, 16) Two or more, (16, 23)
Cheung et al. 2017^70^	83	70 (84)	I1: 68.9 ± 7.7 I2: 74.4 ± 7.5 C: 71.8 ± 8.0	Knee OA	No	None
Fransen et al. 2007^27^	2152	112 (74)	I1: 70.0 ± 6.3 I2: 70.8 ± 6.3 C: 69.6 ± 6.1	Hip/knee OA: % of sample for I1, I2, C Both knees: 74%, 61%, 80% Both hips: 20%, 29%, 29% Single knee: 93%, 73%, 88% Single hip joint: 7%, 27%, 12%	No	OA symptom duration: % of sample for I1, I2, C < 6 years: 31%, 46%, 22% 6–10 years: 35%, 27%, 46% > 10 years: 35%, 25%, 29%
French et al. 2013^71^	131	84 (64)	I1: 61.8 ± 9.7 I2: 64.8 ± 9.8 C: 60.8 ± 9.7	Knee OA	No	Mean number of comorbidities: I1: 2, I2: 2.6, C: 1.86
Hausmann et al. 2017^87^	42	7 (17)	67.5 ± 10.3	% of sample hip/knee/both OA 45%, 55%, 45%	33% of participants had anxiety and 48% depression	Comorbidities mean ± SD: 3.7 ± 2.8
Helminem et al. 2015^88^	111	77 (69)	I: 64.5 ± 7.3 C: 62.8 ± 7.2	Knee OA	No	None
Hurley et al. 2007^77^	418	294 (70)	I: 68 (51–84) C: 67 (51–89)	Knee OA	No	OA symptom duration in years, median (IQR): I: 5 (3–11) C: 6 (3–5)
Kuntz et al. 2018^34^	31	31 (100)	I1: 65.5 ± 5.6 I2: 63.7 ± 9 C: 71.1 ± 9.3	Knee OA	No	Comorbidities mean ± SD: I1: 2.1 ± 1.0 I2: 2.1 ± 1.7 C: 2.9 ± 1.4
Lin et al. 2003^89^	1001	684 (68)	72 ± 7.4	Knee OA	Yes (N, %): Major depression: 159, 16 Dysthymia: 288, 29	None
Lin et al. 2020^72^	80	41 (51)	I: 55.9 ± 15.8 C: 58.1 ± 16.9	Knee OA	No	Comorbidity (N) Yes (52) No (28)
Moonaz et al. 2015^35^	75	72 (96)	52 ± 12	OA (not specified)	No	OA condition duration in years mean ± SD I: 9.9 ± 8.7 C:8.6 ± 9.4
O’Moore et al. 2018^90^	69	55 (80)	I: 63.2 ± 7.4 C: 59.7 ± 6.0	Knee OA	Yes. Major depressive disorder	None
Park et al. 2016^73^	100	75 (75)	75.3 ± 7.5	Lower extremity OA	No	K-L score: 2.4 ± 1.1
Somers et al. 2012^78^	232	184 (79)	58.0 ± 10.4	Knee OA	No	None
Taglietti et al. 2018^36^	60	41 (68)	68.3 ± 4.8	Knee OA	No	None
Tak et al. 2005^79^	109	64 (68)	I: 67.4 ± 7.6 C: 68.9 (SD not provided)	Hip OA	No	None
Walsh et al. 2020^80^	349	216 (62)	I: 66.3 ± 8.1 C: 66.5 ± 8.4	% of sample Hip/knee only I: 57; C: 59 Lower back pain (LBP): I: 59; C: 56 Hip/knee and LBP: I: 65; C: 66	No	None
Yip et al. 2008^81^	95	81 (85)	I: 64.8 ± 10.1 C: 63.4 ± 10.7	Knee OA	No	OA condition duration in years mean ± SD I: 8.0 ± 5.9; C: 6.7 ± 6.0
Zhang et al. 2022^74^	50	37 (74)	I: 55.8 ± 8.4 C: 53.4 ± 10.7	Knee OA	No	None

*C* control, *I* intervention, *K*-*L* Kellgren and Lawrence grading system, *OA* osteoarthritis, *RA* rheumatoid arthritis.

### Meta-analyses

#### Pain

**Figure 2:**
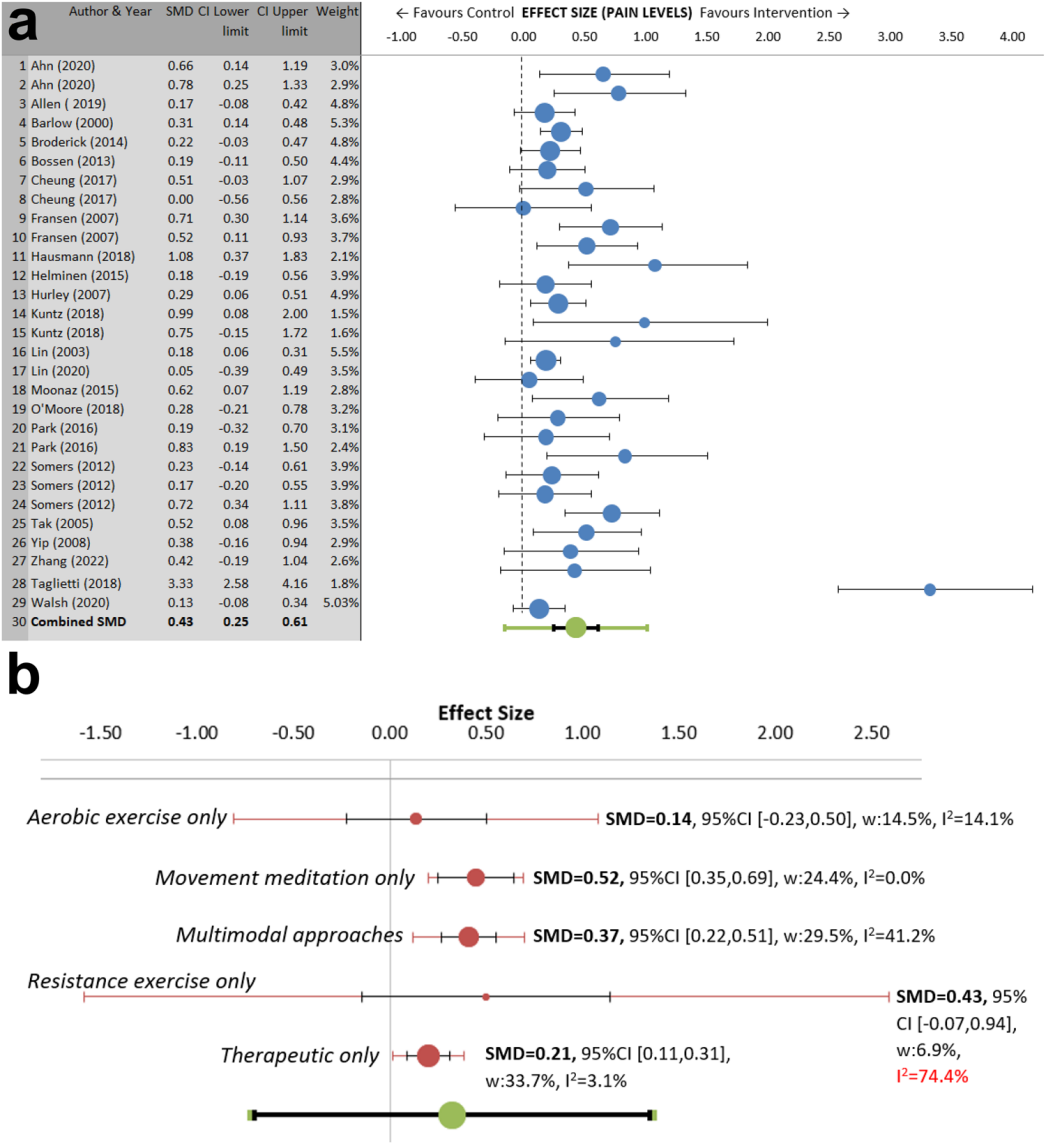
Main meta-analysis (**a**) and sub-group meta-analysis (**b**) for pain. Subgroup meta-analysis results showing effect sizes (i.e. SMDs), CIs (black lines), PIs (red lines) and I^2^ values for interventions. Movement meditation included yoga, tai chi, and qigong. Resistance exercise included hydrotherapy. Multimodal approaches included a combination of exercise and therapeutic/education-based approaches. *CI* confidence interval, I^2^  percentage of variation across studies that is due to heterogeneity, *PI* prediction interval, *SMD* standardized mean difference, *w* weight.

For pain, 29 interventions from 21 published articles were included in the meta-analysis (n = 4,382; 65 ± 6.9 years; 70% female; people with knee/hip OA at varying severity). There was a significant overall medium effect on reducing pain (ES = 0.43, 95% CI [0.25, 0.61], *p* < 0.001). Heterogeneity was significant and substantial (I^2^ = 71.5%, *p*_q_ < 0.001) (Fig. 2a).

Sensitivity analyses were performed to examine potential outliers. A study was excluded with a very large effect size that was in the intervention subtype defined as ‘resistance exercise only’^36^. This analysis included 28 interventions from 20 published articles (n = 4322). The overall effect was ES = 0.33, 95% CI [0.24, 0.42], *p* < 0.001, I^2^ = 32.33%, *p*_q_ = 0.0052. According to Cohen, it is a small to medium effect. Heterogeneity was at an acceptable level.

Subgroup analysis was then performed using random effects models for type of intervention and were defined as aerobic exercise only, resistance exercise only, therapeutic only, multimodal approaches and movement meditation. Effect sizes for reducing pain were significant for movement meditation (7 interventions: ES = 0.52, 95% CI [0.35, 0.69], *p* < 0.001; I^2^ = 0.0%), multimodal approaches (9 interventions: ES = 0.37, 95% CI [0.22, 0.51], *p* < 0.001; I^2^ = 41.2%, *p*_q_ = 0.0092), resistance exercise (2 interventions: ES = 0.43, 95% CI [− 0.07,0.94], *p* = 0.046; I^2^ = 74.4%, *p*_q_ = 0.0048), and therapeutic approaches (7 interventions: ES = 0.21, 95% CI [0.11, 0.31], *p* < 0.001; I^2^ = 3.1%, *p*_q_ = 0.402). Aerobic exercise alone (including 3 interventions) did not significantly improve pain (Fig. 2b). Analysis of variance revealed significant differences between subgroup effect sizes for pain between movement meditation and aerobic exercise (*p* < 0.05) and movement meditation and therapeutic approaches (*p* < 0.01). Heterogeneity was at an acceptable level for all subgroups except resistance exercise though there are too few studies in this group to do further subgroup analysis.

#### Depression

**Figure 3:**
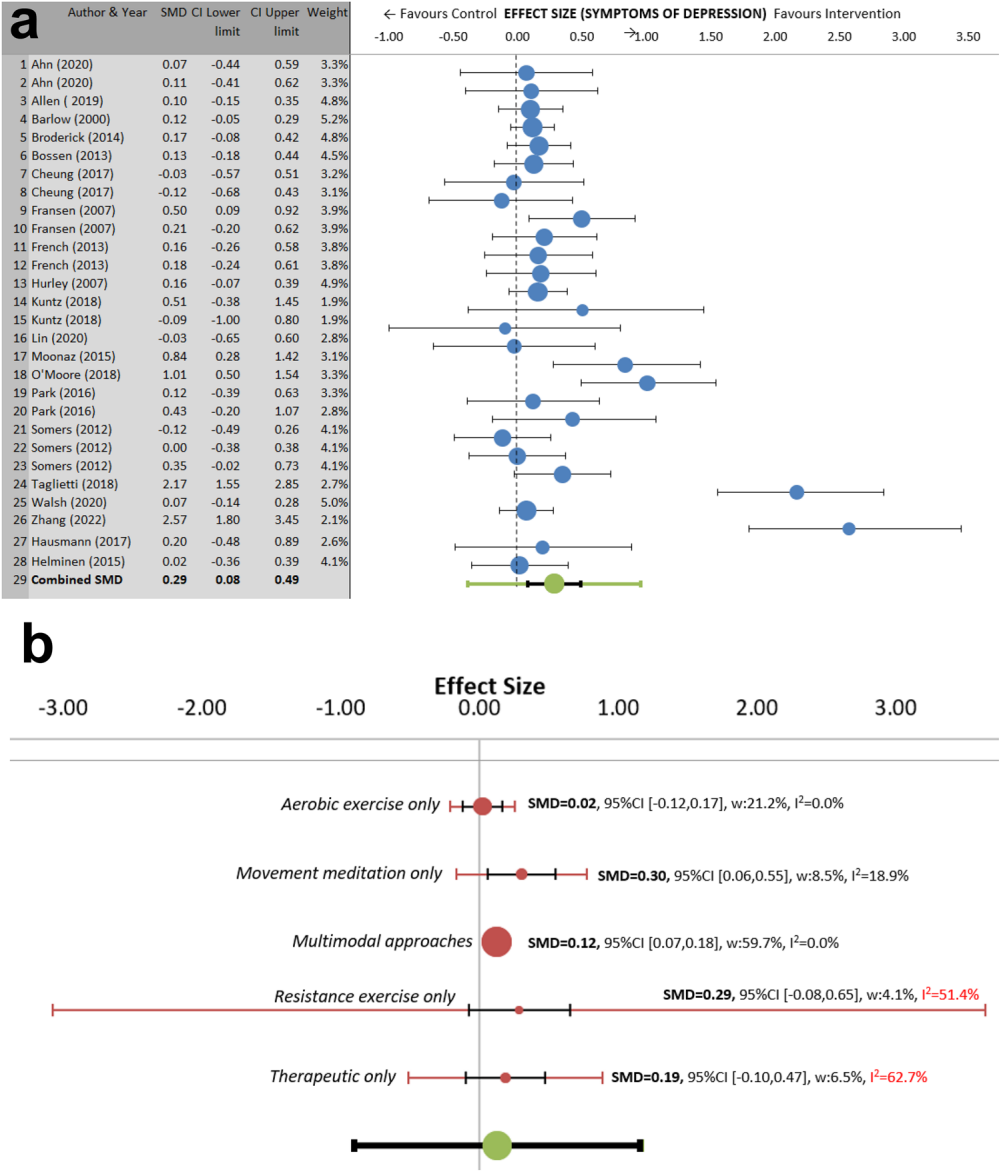
Main meta-analysis (**a**) and sub-group meta-analysis (**b**) for symptoms of depression. Subgroup meta-analysis results showing effect sizes (i.e. SMDs), CIs (black lines), PIs (red lines) and I^2^ values for interventions. Movement meditation included yoga, tai chi, and qigong. Resistance exercise included hydrotherapy. Multimodal approaches included a combination of exercise and therapeutic/education-based approaches. *CI* confidence interval, I^2^ percentage of variation across studies that is due to heterogeneity, *PI* prediction interval, *SMD* standardized mean difference, *w* weight.

For depression, 28 interventions from 20 published articles were included in the meta-analysis (n = 3377; 63 ± 7.0 years; 69% female; people with knee/hip OA at varying severity). There was a significant overall small effect on reducing symptoms of depression (ES = 0.29, 95% CI [0.08, 0.49], *p* < 0.001). Heterogeneity was significant and substantial (I^2^ = 73.3%, *p*_q_ < 0.001) (Fig. 3a).

For the sensitivity analysis two studies were excluded with very large effect sizes. They included a resistance intervention^36^ and a movement meditation intervention^74^. This analysis included 26 interventions from 18 published articles (n = 3274). The overall effect is small and significant ES = 0.16, 95% CI [0.08, 0.24], *p* < 0.001, I^2^ = 11.78%, *p*_q_ = 0.293. Heterogeneity was at an acceptable level.

Subgroup analysis was then performed using random effects models for type of intervention and were defined as aerobic exercise only, resistance exercise only, therapeutic only, multimodal approaches and movement meditation. Effect sizes for reducing symptoms of depression were significant for movement meditation (6 interventions: ES = 0.30, 95% CI [0.06, 0.55], *p* = 0.008, I^2^ = 18.9%, *p*_q_ = 0.291), and multimodal interventions (8 interventions: ES = 0.12, 95% CI [0.07, 0.18], *p* < 0.001, I^2^ = 0%, *p*_q_ = 0.940). Resistance exercise, aerobic exercise, or therapeutic approaches alone (2, 4 and 6 interventions respectively) did not improve symptoms of depression (Fig. 3b). Analysis of variance revealed no significant differences between subgroup effect sizes for depression. Heterogeneity was at an acceptable level for movement meditation, multimodal approaches and aerobic exercise though was high for the remaining subgroups.

#### Publication bias

**Figure 4:**
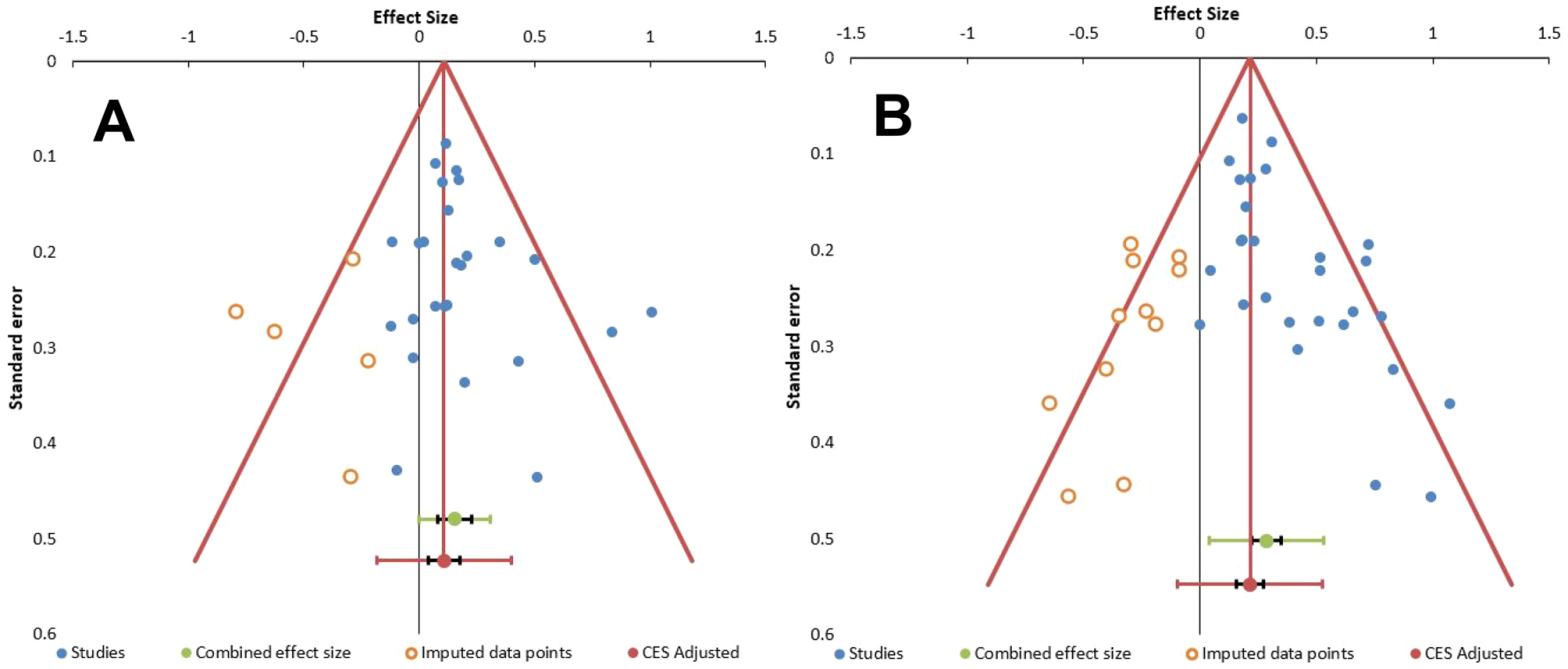
Funnel plot of all included studies for (**A**) pain, and (**B**) symptoms of depression.

A funnel plot and the Egger test of asymmetry were used to examine publication bias (Fig. 4). The funnel plot was asymmetric. The Egger test was significant for pain (*p* < 0.001) suggesting potential publication bias and/or small-study effects^91^, though was not significant for depression (*p* = 0.216).

## Discussion

### Summary of main findings

Overall, meta-analyses from 23 high quality rated studies showed that nonpharmacological approaches significantly reduced pain (ES = 0.43, 95% CI [0.25, 0.61], 29 interventions) and symptoms of depression (ES = 0.29, 95% CI [0.08, 0.49], 28 interventions) in people with OA. Heterogeneity was lowered after removing outliers. For pain, subgroup analysis showed significance for movement meditation, multimodal approaches, therapeutic approaches, and resistance exercise only. However, heterogeneity was considerably high for resistance exercise. Movement meditation and multimodal approaches were also significant for symptoms of depression.

Based on these findings, approaches that are either multimodal (e.g. education/therapeutic approaches alongside exercise) or include focus on both the body and mind (e.g. focused breathing and mindfulness techniques used alongside movement, seen in yoga, qigong, and tai chi) are effective for reducing pain and symptoms of depression in people with knee/hip OA and warrant further investigation.

### How these findings compare to others

Our findings support and expand on others regarding beneficial effects of exercise for reducing pain in people living with OA^23,87^. There are also differences between our findings and those of other studies. Fransen et al.’s^26^ Cochrane review based on 44 trials indicated that exercise significantly reduced pain and improved physical function^26^. Uthman et al.’s^92^ review based on 60 trials compared the effectiveness of different exercise interventions^92^.

Our analysis included only two resistance and three aerobic interventions. Both Cochrane reviews included more articles than our review. This was likely due to the main outcome of interest being pain and did not include depression, and because we only included studies with an inactive control group. Our findings align with other studies regarding reducing pain^26,92^, though extend our current understanding by investigating exercise type. In Fransen et al.’s review, no subgroup analysis was performed to examine potential different effects of exercise type, despite the heterogeneity and marked variability that was present regarding the exercise interventions assessed and other aspects of methodology^26^.

A more recent network meta-analysis investigated the relative efficacy of different types of exercise (aerobic, mind–body, strengthening, flexibility/skill or mixed) and found that aerobic had an equivalent beneficial effect (SMD = 1.11) to mind–body approaches in reducing pain^93^. Additionally, reporting that mixed exercise was the least effective. However, these approaches did not include education/therapeutic components as we did here, nor did they examine depression symptomatology. In another network analysis that examined different approaches on mental health measures in people with OA, the authors concluded that strengthening exercise was most beneficial for overall mental health, or mixed exercise for symptoms of depression^94^, though pain was not examined.

### Strengths and limitations

Strengths of this study include that we were able to perform a meta-analysis including 23 studies where one or more intervention groups were compared with a control group. We also achieved high agreement levels between independent reviewers on ratings of study quality. We showed that similar approaches were effective for reducing both pain and symptoms of depression in OA, suggesting that these benefits will likely transfer to additional improvements in quality of life^92,90^. Further, identifying effective therapeutic options can support informed choice for people living with OA, and thus support shared decision-making processes in collaboration with healthcare professionals and family members to tailor treatments that best suit their individual needs and preferences^95^.

However, there are several limitations to consider when interpreting these results. Although the overall effect obtained for the meta-analyses for both pain and depression were significant, not all studies reported beneficial effects (e.g. pain^72,85^, symptoms of depression^79,89^). Possible explanations for observed non-significant effects in individual studies include low intervention dose, baseline depression scores and/or lack of formal depression diagnoses, and that participants differed between studies in the diagnostic site of OA, e.g. knee or hip. In our study, this information is included in Tables 1 and 2 where available.

Publication bias should also be considered, and some studies had very small sample sizes which may increase the likelihood of Type II errors. The funnel plots and Egger’s regression indicated publication bias was likely for pain level data though not for symptoms of depression, possibly due to pain more frequently being recorded as a primary outcome measure and depression a secondary outcome measure in these studies. Therefore, the overall effect size for pain may be an overestimation. This can be caused when small sample studies have not been published because significant effects were not obtained.

We included RCTs that measured pain and symptoms of depression in OA. Studies not measuring depression though were effective in reducing pain in OA may not have been captured in this review, for example therapeutic exercise and pain education^96^. Safety is a key consideration in intervention studies additional to effectiveness and adverse effects have been captured in other reviews^26,97^. However, we did not extract data for this outcome. Exercise is usually safe and well-tolerated for people with OA and depression, but this is based on limited data as studies seldom collect this as an outcome. Future trials would benefit from more focus and rigorous reporting on safety.

### Clinical implications

In this systematic review we provide evidence for several approaches for reducing pain and symptoms of depression in OA, a condition with a substantial health burden in which co-morbidities are common. We highlight the importance of considering psychological health, i.e. symptoms of depression, in addition to physical health, i.e. pain, when providing quality healthcare to people living with OA. Interventions may be more effective when they are tailored to the individual and involve healthcare professionals working collaboratively across disciplines, e.g. nutrition, exercise, psychology, as well as with patients/clients. Further investigation is warranted on combining psychological and physical health focused interventions as well as multidisciplinary expertise to optimize patient outcomes.

Pain is experienced by at least half of people living with dementia and is most often attributed to OA^98^. Given the strong associations between OA and dementia^6–8, 98^*,* and that forty percent of dementia cases could be prevented or delayed by targeting risk factors including physical inactivity and associated chronic conditions^31,99^, targeting associated chronic conditions including OA will likely lead to reductions in dementia prevalence rates.

## Conclusion

In this study, mind–body approaches were more effective than aerobic/resistance exercise or psychological therapy alone for reducing pain and depression in people with OA. Future research is required to determine the optimal approach for OA based on diagnostic site and symptom severity of comorbid conditions such as depression. Further, given the high prevalence rates of chronic conditions and people who experience multimorbidity, a better understanding of how the underlying mechanisms of co-occurring chronic conditions interact is also required. Incorporating qualitative studies such as focus groups and interviews to examine the experience, perspectives and preferences of people living with OA and depression will also add considerable value to this area.

### Supplementary Information

Supplementary Information.

## Data Availability

The data will be made available on request to the corresponding author.
