# Supplementary material for: Nonpharmacological approaches for pain and symptoms of depression in people with osteoarthritis: systematic review and meta-analyses
Source: Sci Rep. 2023 Sep 18;13:15449. doi: 10.1038/s41598-023-41709-x (PMC10507102; doi:10.1038/s41598-023-41709-x)
Supplement: Supplementary file 1 — Supplementary Information. [file 41598_2023_41709_MOESM1_ESM.pdf]

Nonpharmacological approaches for pain and symptoms of depression in people with osteoarthritis: Systematic review and meta-analyses.

*Claire V Burley[a,b] , Anne-Nicole Casey[b], Matthew Jones[a,c] , Kemi E Wright[a], and Belinda J Parmenter[a]
[a] *School of Health Sciences, University of New South Wales, Sydney, Australia*
[b] *Centre for Healthy Brain Ageing, School of Clinical Medicine, University of New South Wales, Sydney, Australia*
[c] *Centre for Pain IMPACT, Neuroscience Research Australia, Sydney, Australia*

*Corresponding author:
Dr Claire V Burley
UNSW Lifestyle Clinic, Level 2, Wallace Wurth Building
School of Health Sciences, Faculty of Medicine & Health
University of New South Wales
Sydney NSW 2052 AUSTRALIA
c.burley@unsw.edu.au

Supplementary Material

S1. Complete list of database search terms.

History and Search Details — Download — Delete

Search	Actions	Details	Query	Results	Time
#8	•••	>	Search: **#1 AND #2 AND #3 AND #4**	539	00:30:28
#7	•••	>	Search: **#1 AND #2 AND #4**	592	00:29:29
#6	•••	>	Search: **#1 AND #2 AND #3**	4,494	00:28:47
#5	•••	>	Search: **#1 AND #2**	7,504	00:28:20
#4	•••	>	Search: **RCT OR "Randomised controlled trial" OR "Randomized controlled trial" OR "randomised controlled trial" OR "Randomized controlled trial"**	625,120	00:25:52
#3	•••	>	Search: **Exercise OR "physical activity" OR education OR therapy OR CBT OR "cognitive behavioural" OR cognitive-behavioural OR "lifestyle intervention"**	12,204,142	00:24:12
#2	•••	>	Search: **Depression OR anxiety OR mood OR affective OR psychological**	2,024,322	00:23:24
#1	•••	>	Search: **Osteoarthritis OR OA**	122,993	00:22:53

Showing 1 to 8 of 8 entries

Search: **#1 AND #2 AND #3 AND #4**
("osteoarthritis"[MeSH Terms] OR "osteoarthritis"[All Fields] OR "osteoarthritides"[All Fields] OR "OA"[All Fields]) AND ("depressed"[All Fields] OR "depression"[MeSH Terms] OR "depression"[All Fields] OR "depressions"[All Fields] OR "depression s"[All Fields] OR "depressive disorder"[MeSH Terms] OR ("depressive"[All Fields] AND "disorder"[All Fields])

OR "depressive disorder"[All Fields] OR "depressivity"[All Fields] OR "depressive"[All Fields] OR "depressively"[All Fields] OR "depressiveness"[All Fields] OR "depressives"[All Fields] OR ("anxiety"[MeSH Terms] OR "anxiety"[All Fields] OR "anxieties"[All Fields] OR "anxiety s"[All Fields]) OR ("affect"[MeSH Terms] OR "affect"[All Fields] OR "mood"[All Fields]) OR ("affect s"[All Fields] OR "affectional"[All Fields] OR "affective"[All Fields] OR "affectively"[All Fields] OR "affectives"[All Fields] OR "affectivity"[All Fields]) OR ("psychologic"[All Fields] OR "psychological"[All Fields] OR "psychologically"[All Fields] OR "psychologization"[All Fields] OR "psychologized"[All Fields] OR "psychologizing"[All Fields])) AND ("exercise"[MeSH Terms] OR "exercise"[All Fields] OR "exercises"[All Fields] OR "exercise therapy"[MeSH Terms] OR ("exercise"[All Fields] AND "therapy"[All Fields]) OR "exercise therapy"[All Fields] OR "exercise s"[All Fields] OR "exercised"[All Fields] OR "exerciser"[All Fields] OR "exercisers"[All Fields] OR "exercising"[All Fields] OR "physical activity"[All Fields] OR ("educability"[All Fields] OR "educable"[All Fields] OR "educates"[All Fields] OR "education"[MeSH Subheading] OR "education"[All Fields] OR "educational status"[MeSH Terms] OR ("educational"[All Fields] AND "status"[All Fields]) OR "educational status"[All Fields] OR "education"[MeSH Terms] OR "education s"[All Fields] OR "educational"[All Fields] OR "educative"[All Fields] OR "educator"[All Fields] OR "educator s"[All Fields] OR "educators"[All Fields] OR "teaching"[MeSH Terms] OR "teaching"[All Fields] OR "educate"[All Fields] OR "educated"[All Fields] OR "educating"[All Fields] OR "educations"[All Fields]) OR ("therapeutics"[MeSH Terms] OR "therapeutics"[All Fields] OR "therapies"[All Fields] OR "therapy"[MeSH Subheading] OR "therapy"[All Fields] OR "therapy s"[All Fields] OR "therapys"[All Fields]) OR "CBT"[All Fields] OR "cognitive-behavioural"[All Fields] OR "cognitive-behavioural"[All Fields] OR "lifestyle intervention"[All Fields] OR ("mind s"[All Fields] OR "minded"[All Fields] OR "mindful"[All Fields] OR "mindfulness"[MeSH Terms] OR "mindfulness"[All Fields] OR "minding"[All Fields] OR "minds"[All Fields])) AND ("RCT"[All Fields] OR "Randomised controlled trial"[All Fields] OR "Randomized controlled trial"[All Fields] OR "Randomised controlled trial"[All Fields] OR "Randomized controlled trial"[All Fields])

Translations

Osteoarthritis: "osteoarthritis"[MeSH Terms] OR "osteoarthritis"[All Fields] OR "osteoarthritides"[All Fields]

Depression: "depressed"[All Fields] OR "depression"[MeSH Terms] OR "depression"[All Fields] OR "depressions"[All Fields] OR "depression's"[All Fields] OR "depressive disorder"[MeSH Terms] OR ("depressive"[All Fields] AND "disorder"[All Fields]) OR "depressive disorder"[All Fields] OR "depressivity"[All Fields] OR "depressive"[All Fields] OR "depressively"[All Fields] OR "depressiveness"[All Fields] OR "depressives"[All Fields]

anxiety: "anxiety"[MeSH Terms] OR "anxiety"[All Fields] OR "anxieties"[All Fields] OR "anxiety's"[All Fields]

mood: "affect"[MeSH Terms] OR "affect"[All Fields] OR "mood"[All Fields]

affective: "affect's"[All Fields] OR "affectional"[All Fields] OR "affective"[All Fields] OR "affectively"[All Fields] OR "affectives"[All Fields] OR "affectivity"[All Fields]

psychological: "psychologic"[All Fields] OR "psychological"[All Fields] OR "psychologically"[All Fields] OR "psychologization"[All Fields] OR "psychologized"[All Fields] OR "psychologizing"[All Fields]
Exercise: "exercise"[MeSH Terms] OR "exercise"[All Fields] OR "exercises"[All Fields] OR "exercise therapy"[MeSH Terms] OR ("exercise"[All Fields] AND "therapy"[All Fields]) OR "exercise therapy"[All Fields] OR "exercise's"[All Fields] OR "exercised"[All Fields] OR "exerciser"[All Fields] OR "exercisers"[All Fields] OR "exercising"[All Fields]
education: "educability"[All Fields] OR "educable"[All Fields] OR "educates"[All Fields] OR "education"[Subheading] OR "education"[All Fields] OR "educational status"[MeSH Terms] OR ("educational"[All Fields] AND "status"[All Fields]) OR "educational status"[All Fields] OR "education"[MeSH Terms] OR "education's"[All Fields] OR "educational"[All Fields] OR "educative"[All Fields] OR "educator"[All Fields] OR "educator's"[All Fields] OR "educators"[All Fields] OR "teaching"[MeSH Terms] OR "teaching"[All Fields] OR "educate"[All Fields] OR "educated"[All Fields] OR "educating"[All Fields] OR "educations"[All Fields]
therapy: "therapeutics"[MeSH Terms] OR "therapeutics"[All Fields] OR "therapies"[All Fields] OR "therapy"[Subheading] OR "therapy"[All Fields] OR "therapy's"[All Fields] OR "therapys"[All Fields]
mindfulness: "mind's"[All Fields] OR "minded"[All Fields] OR "mindful"[All Fields] OR "mindfulness"[MeSH Terms] OR "mindfulness"[All Fields] OR "minding"[All Fields] OR "minds"[All Fields]

S2. Quality rating sheet.

Criterion		Score
**Design**		
1	Randomised	
2	Randomised according to Delphi specifications i.e., must be unpredictable e.g., coin toss, table of random numbers, computer generated etc. *(by DOB, admission date, MRN, coin toss of clusters or similar not OK)*	
3	Control or comparison group *(repeated measures OK)*	
4	Blinded ratings *(partial blinding OK if primary outcome blinded)*	
**Participants**		
5	Groups similar at baseline regarding most important prognostic indicators *(≤20% difference OK. Must include age, gender & baseline "behavior" score or an indication that there is no significant difference in these. Where groups are not matched but baseline 'behavior' scores are used as a covariate in analysis – OK. Repeated measures OK. Behavior change score only not OK)*	
6	Eligibility/inclusion criteria specified *(could the study be replicated based on the information reported?)*	
7	Use of standardized diagnostic criteria e.g., GDS, MMSE, DSM, ICD, etc. *(no credit where criteria not reported, 'written in notes by dr' or 'diagnosed by dr', etc.*	
8	All subjects accounted for/withdrawals noted	
**Outcomes**		
9	Well-validated, reliable measures *(known or reported as validated, published generally OK)*	
10	Objective outcome *(based on observations OK, not self-rated)*	
11	Follow-up assessment 6 months or beyond (i.e., from **cessation** of intervention)	
**Statistics**		
12	Point estimates and measures of variability provided for primary outcome measures *(both means + SDs or SEs or effect sizes provided)*	
13	Statistical significance considered and reported	
14	Adjustment for multiple comparisons, e.g., adjusted p-value, Bonferroni, Scheffe, Tukey's, post hoc, hierarchical linear modelling *(no credit where not reported)*	
15	Evidence of sufficient power ≥ 80% *(reported or large sample size, i.e., n ≈ 100)*	
16	Intention-to-treat analysis included i.e., **all** randomized participants included in analysis *(not just stated as ITT)* or<br>non-randomized study - **all** participants enrolled are included in analysis	
**Total score**		
Quality rating: modest (1-5) moderate (6-10) strong (11-16)		
Effect size: insufficient info small (0.2) medium (0.5) large (0.8)		
Comments/queries		
